# Supplementary material for: A conserved YAP/Notch/REST network controls the neuroendocrine cell fate in the lungs
Source: Nat Commun. 2022 May 16;13:2690. doi: 10.1038/s41467-022-30416-2 (PMC9110333; doi:10.1038/s41467-022-30416-2)
Supplement: Supplementary file 1 — Supplementary Information [file 41467_2022_30416_MOESM1_ESM.pdf]

# Supplementary Figure 1

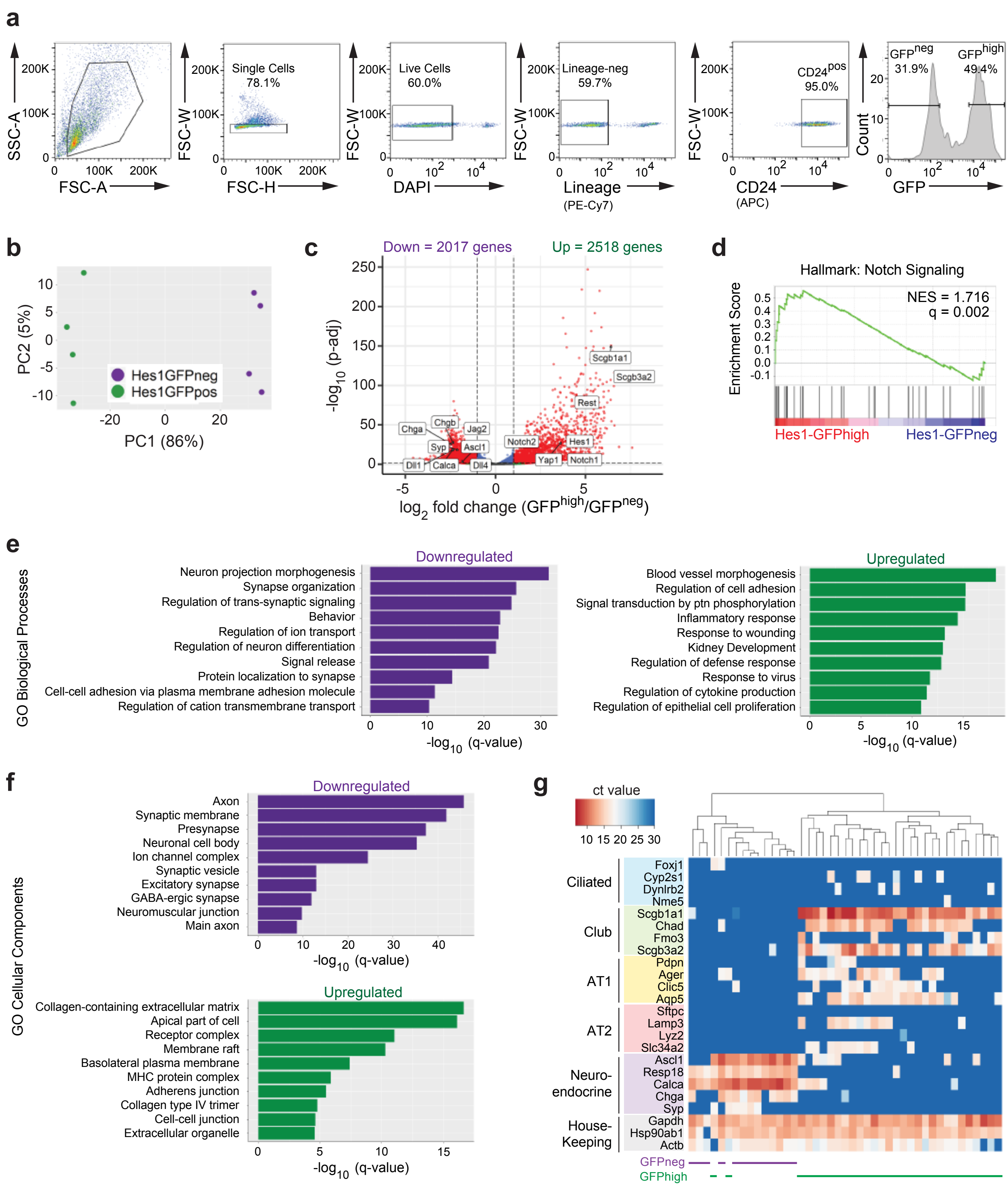


**Supplementary Figure 1: Characterization of HES1-positive non-neuroendocrine SCLC cells**

**a,** Flow cytometry strategy to isolate GFP$^{high}$ and GFP$^{neg}$ SCLC cells from *Rb/p53/p130/Hes1$^{GFP/+}$* mutant mouse SCLC tumors. **b,c,** Principal component analysis (PCA) (b) of RNA-seq data comparing GFP$^{high}$ and GFP$^{neg}$ SCLC cells (n=4 paired samples, each pair sorted from different mice). Volcano plot (c) of differentially expressed genes from the RNA-seq data. Classical neuroendocrine/non-neuroendocrine markers and Notch pathway members are highlighted (significant genes with more than 2-fold change and p-adj value < 0.05 are in red). Wald test with Benjamini-Hochberg correction. **d,** Gene set enrichment analysis (GSEA) for Notch pathway signature enriched in GFP$^{high}$ cells compared to GFP$^{neg}$ cells (from MSigDB Hallmark gene sets). **e,f,** Gene ontology (GO) enrichment comparing GFP$^{high}$ (green) and GFP$^{neg}$ (purple) SCLC cells for Biological Processes (e) and Cellular Components (f). **g,** Independent replicate of single-cell RT-qPCR analysis (Fluidigm) of GFP$^{high}$ and GFP$^{neg}$ SCLC cells for markers of lung epithelial cells (n=43 cells). Source data are provided as a Source Data file.

## Supplementary Figure 2

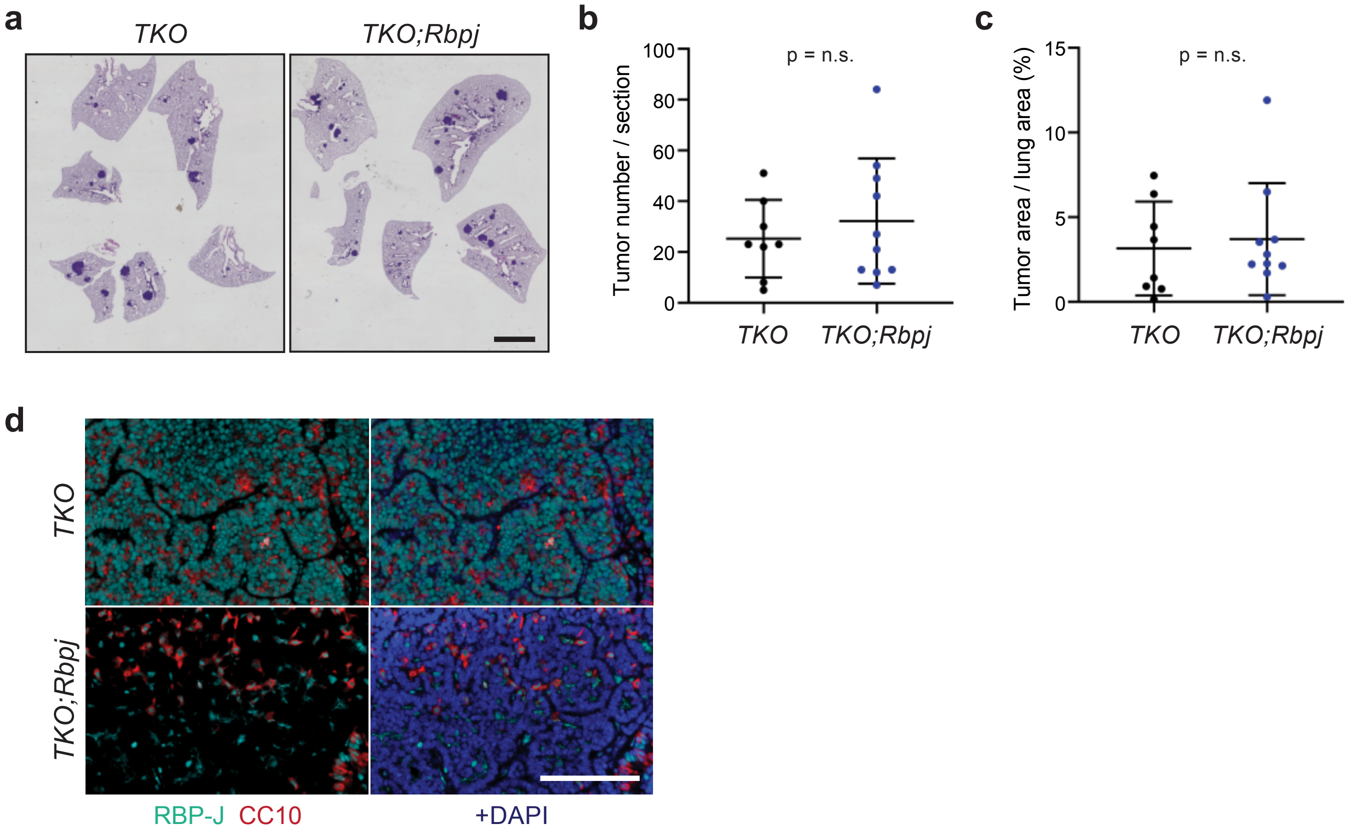


**Supplementary Figure 2: Deletion of *Rbpj* in the *TKO* mouse model does not affect tumor burden but blocks transdifferentiation to the non-NE state.**

**a-c,** (a) Representative images of lung sections from *TKO* and *TKO;Rbpj*$^{fl/fl}$ mice 5 months after Ad-CMV-Cre (*TKO*;*Rbpj*, quadruple mutant), stained with hematoxylin and eosin (H&E). Scale bar, 3 mm. Quantification of tumor numbers (b) and tumor area (c) (n=8 mice for *TKO* and 10 mice for *TKO;Rbpj*$^{fl/fl}$). Unpaired t-test, data represented as mean ± s.d. **d,** Representative immunofluorescence images for RBP-J (cyan) and CC10 (red, encoded by *Scgb1a1*) on a tumor section from either *TKO* or *TKO;Rbpj*$^{fl/fl}$ mice. DAPI stains the DNA in blue. Scale bar, 100 µm. Source data are provided as a Source Data file.

## Supplementary Figure 3

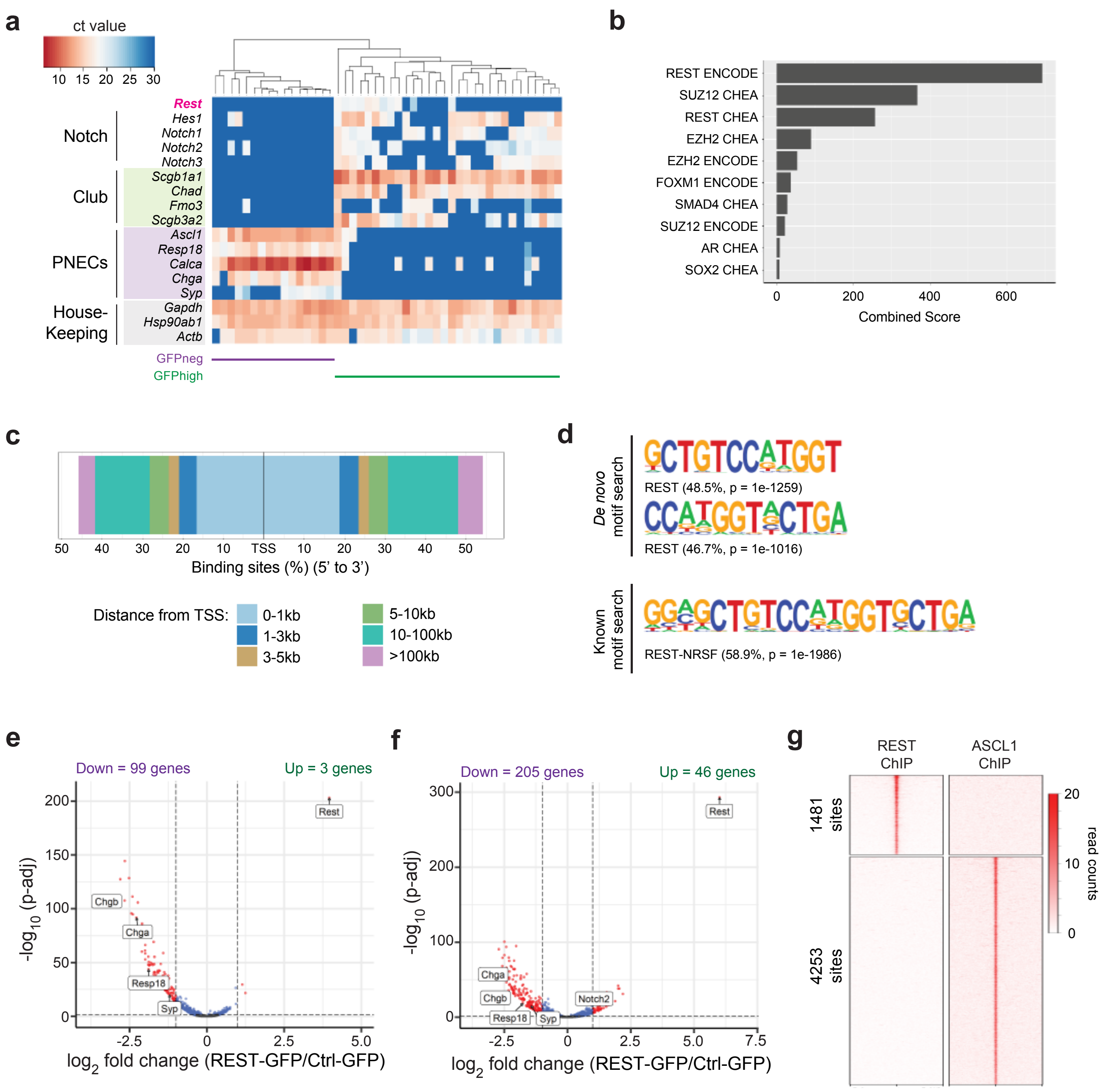


**Supplementary Figure 3: REST and ASCL1 have non-overlapping binding sites in SCLC cells**

**a,** Single-cell RT-qPCR analysis (Fluidigm) of GFP$^{high}$ and GFP$^{neg}$ SCLC cells isolated from *TKO;Hes1*$^{GFP/+}$ mutant mouse SCLC tumors for *Rest*, Notch pathway members, club cell and PNEC markers (n=46 cells). **b,** Bar graph showing top 10 candidate transcriptional factors from Enrichr analysis (ENCODE and ChEA Consensus TFs from ChIP-X) of the downregulated genes in GFP$^{high}$ SCLC cells compared to GFP$^{neg}$ cells from *TKO;Hes1*$^{GFP/+}$ tumors. **c,** Distribution of REST binding sites upstream and downstream of the transcription start site (TSS) of the nearest gene from ChIP-seq analysis in GFP$^{high}$ cells in culture. The majority of the binding sites are within a few kb of TSS. **d,** Top DNA motifs found in REST-bound regions by HOMER analysis. **e,f,** Volcano plot of differentially expressed genes from RNA-seq data of 48 hours (e) and 5 days (f) overexpression of REST in mouse KP1 neuroendocrine SCLC cells. *Rest*, *Notch2* and classical neuroendocrine markers are highlighted (significant genes with more than 2-fold change and p-adj value < 0.05 are in red). Wald test with Benjamini-Hochberg correction. **g,** ChIP-seq signal for REST and ASCL1 in mouse SCLC tumor cells quantified ± 5 kb around peak summits. Source data are provided as a Source Data file.

# Supplementary Figure 4

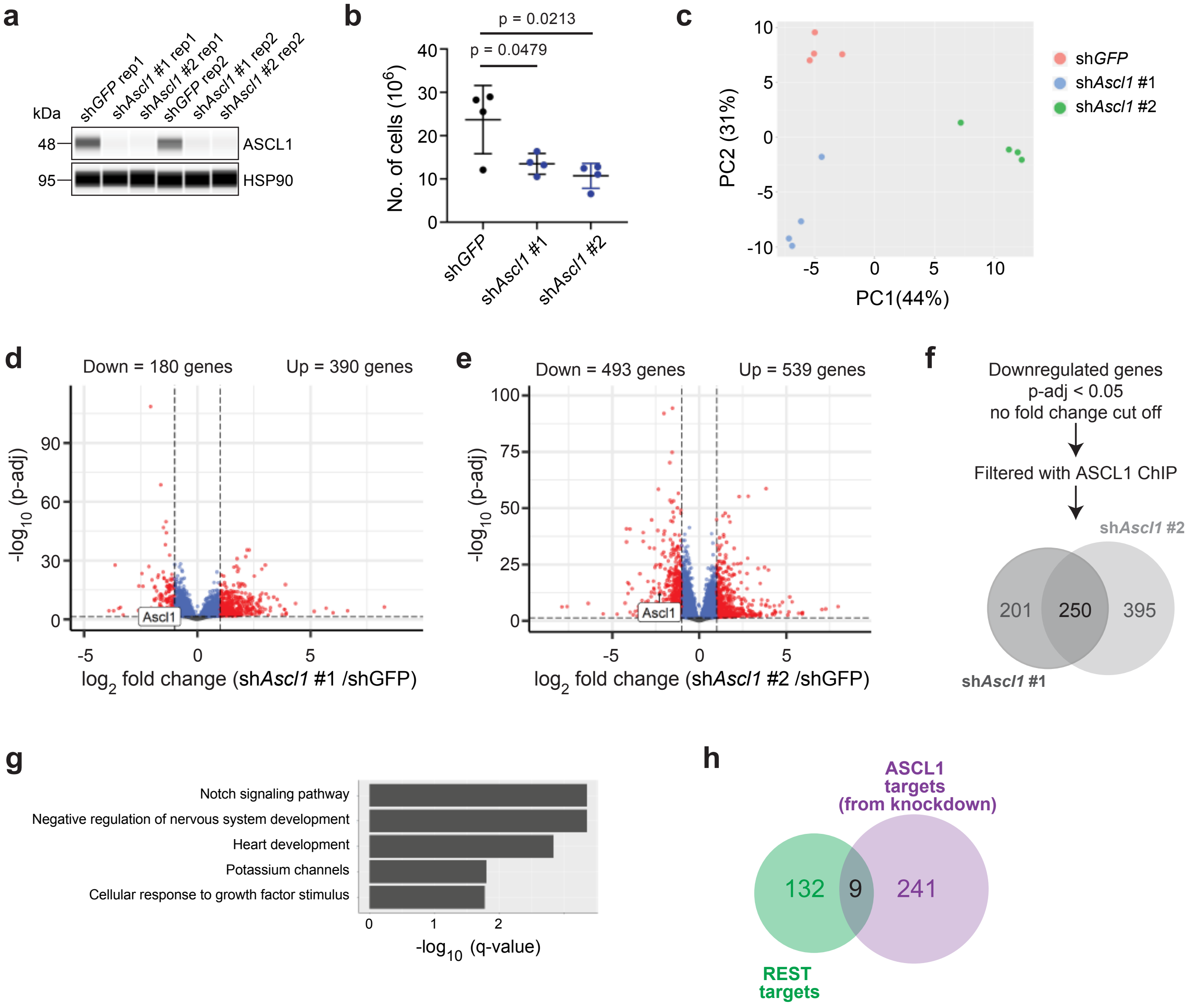


**Supplementary Figure 4: ASCL1 targets identified via *Ascl1* knockdown in KP1 cells show limited overlap with REST targets**

**a-c,** (a) Immunoassay for ASCL1 (samples from two independent replicates), (b) cell counting assay (n=4 independent rounds of lentivirus production and transduction, unpaired t-test, data represented as mean ± s.d.) and (c) principal component analysis (PCA) of RNA-seq data of mouse KP1 neuroendocrine SCLC cells 3 days after lentiviral transduction of shGFP or sh*Ascl1* (#1 and #2 represents two different hairpins). **d-e,** Volcano plot of differentially expressed genes from the RNA-seq data comparing each hairpin (d) sh*Ascl*1 #1 or (e) sh*Ascl1* #2 to sh*GFP* control (significant genes with more than 2-fold change and p-adj value < 0.05 are in red). Wald test with Benjamini-Hochberg correction. **f,** Experimental workflow to identify ASCL1 candidate targets from the *Ascl1* knockdown experiments. **g,** Gene ontology (GO) analysis of the 250 ASCL1 candidate targets identified in (f). **h,** Venn diagram showing the degree of overlap between ASCL1 candidate targets identified in (f) and REST targets identified in main figure 2(a). See also Suppl. Data 7 and 8. Source data are provided as a Source Data file.

# Supplementary Figure 5

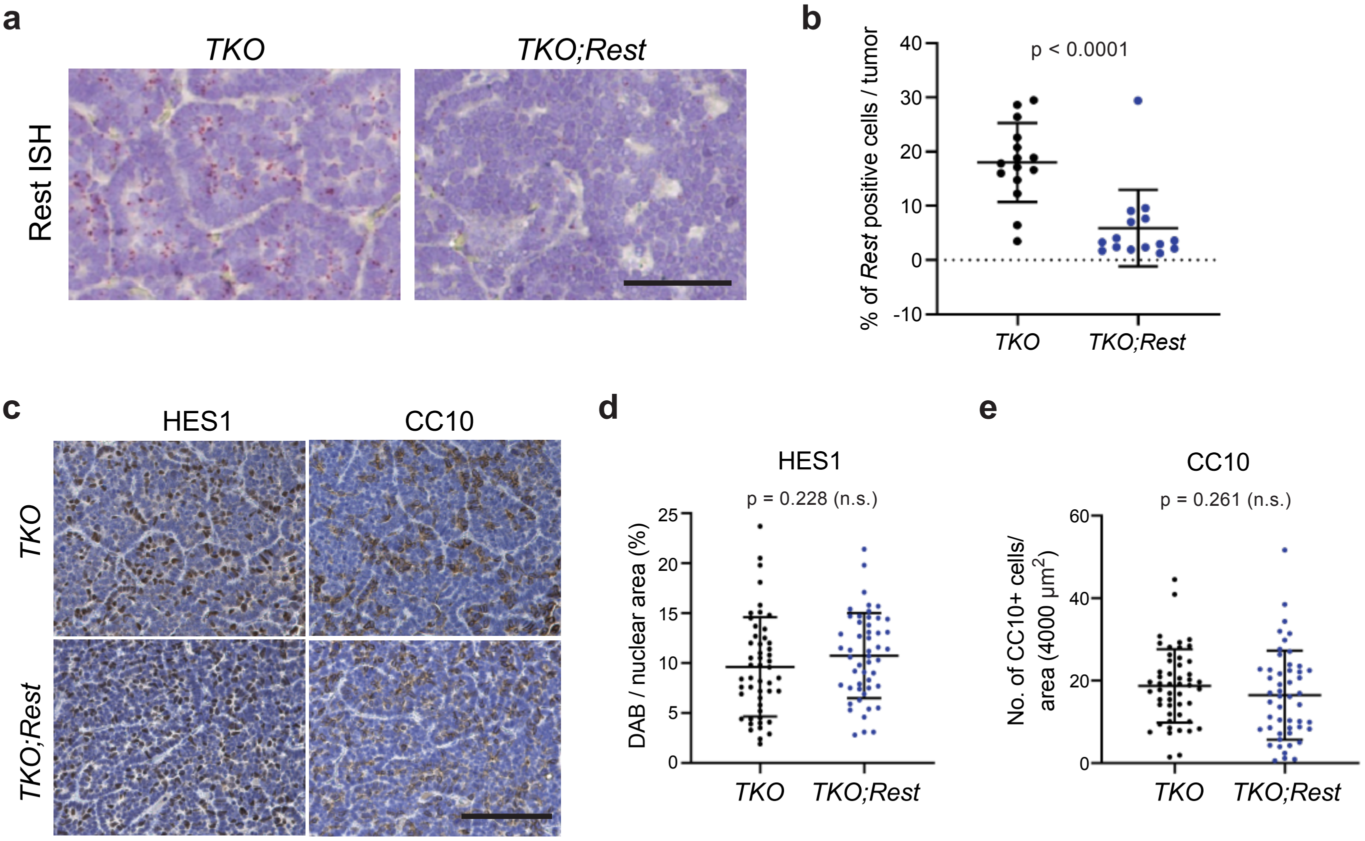


**Supplementary Figure 5: Loss of REST does not prevent upregulation of HES1 and CC10 in non-neuroendocrine SCLC cells**

**a,** Representative brightfield images of *Rest* RNAscope *in situ* hybridization (ISH, red signal) in *TKO* and *TKO;Rest* mutant tumors (hematoxylin counterstain). Scale bar, 50 µm. **b,** Quantification of *Rest* RNAscope ISH in *TKO* and *TKO;Rest* mutant tumors in (a) (n=5 tumors/mouse from 3 mice/genotype). Unpaired t-test, data represented as mean ± s.d. **c-e,** (c) Representative immunohistochemistry (IHC) images of HES1 and CC10 staining (brown signal) in *TKO* and *TKO;Rest* mutant tumors (hematoxylin counterstain). Scale bar, 100 µm. Quantification of HES1 DAB staining (d) and CC10 DAB staining (e) in tumor sections (n=10 tumors/mouse from 5 mice/group). Unpaired t-test, data represented as mean ± s.d. Source data are provided as a Source Data file.

# Supplementary Figure 6

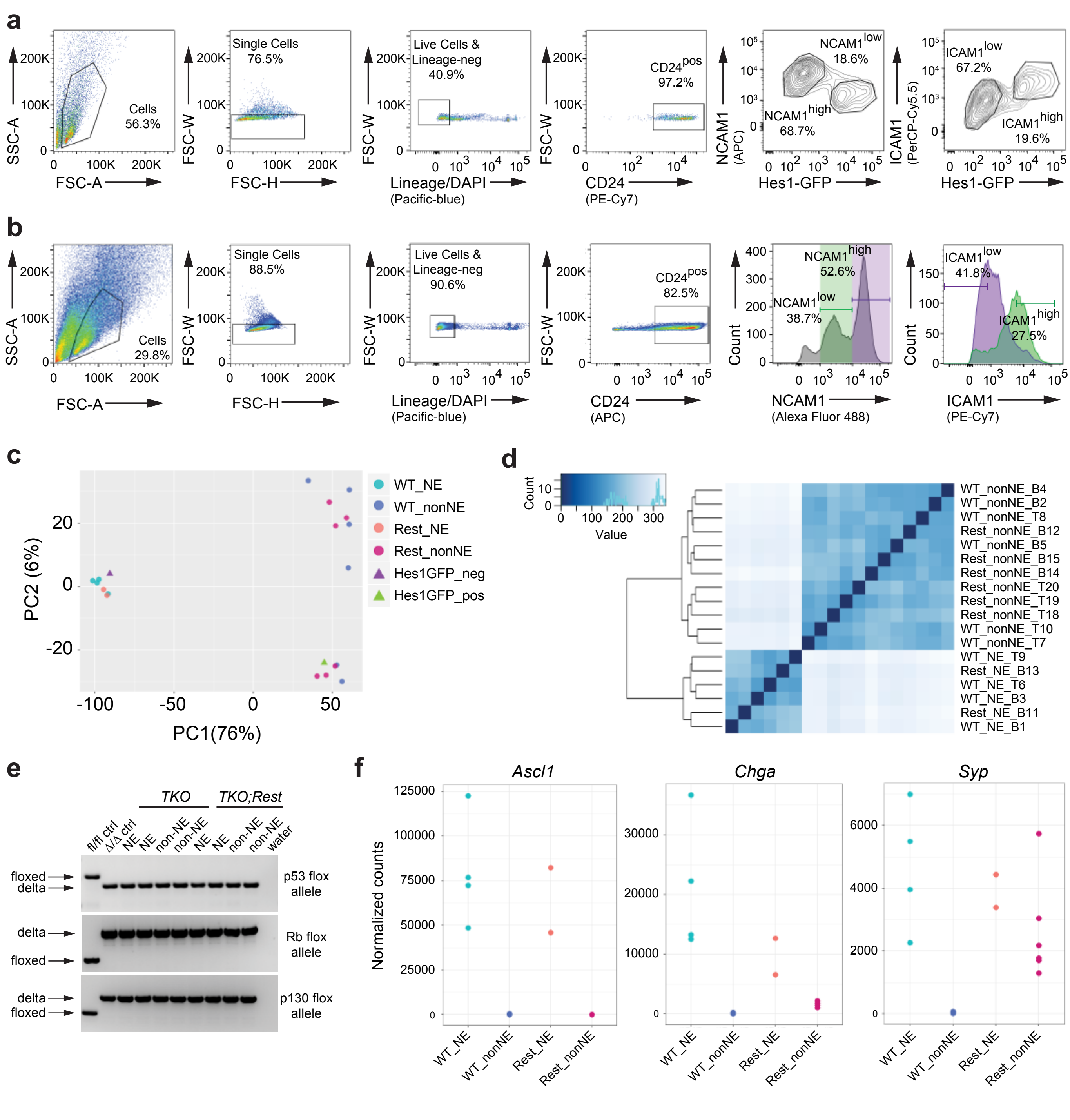

**Supplementary Data Figure 6: Loss of REST in SCLC cells prevents full transition from the neuroendocrine to the non-neuroendocrine state**

**a,** Flow cytometry strategy identifying NCAM1 and ICAM1 as cell surface markers to isolate NE and non-NE SCLC cells from *TKO;Hes1*$^{GFP/+}$ SCLC tumors. **b,** Flow cytometry strategy to isolate NE and non-NE SCLC cells from *TKO* and *TKO;Rest* mutant SCLC tumors. **c,d,** PCA (c) and hierarchical clustering (d) of RNA-seq data comparing NE and non-NE cell lines generated from *TKO* and *TKO;Rest* mutant tumors (n=4 WT_NE, 6 WT_nonNE, 2 Rest_NE, 6 Rest_nonNE). WT, wild-type for *Rest*; "Rest", knockout for *Rest*. Refer to Suppl. Data 15 for complete sample list, B = derived from bulk/pooled tumors and T = derived from single tumor. **e,** Genotyping PCR analysis for recombined (delta, Δ) or unrecombined (floxed) alleles at the *p53*, *Rb*, and *p130* loci in NE and non-NE cells sorted from tumors in *TKO* and *TKO;Rest*$^{fl/fl}$ mice. Control (ctrl) DNAs are indicated. **f,** Normalized counts graphs from the RNA-seq data for *Ascl1*, *ChgA* and *Syp* across all samples listed in (d).

# Supplementary Figure 7

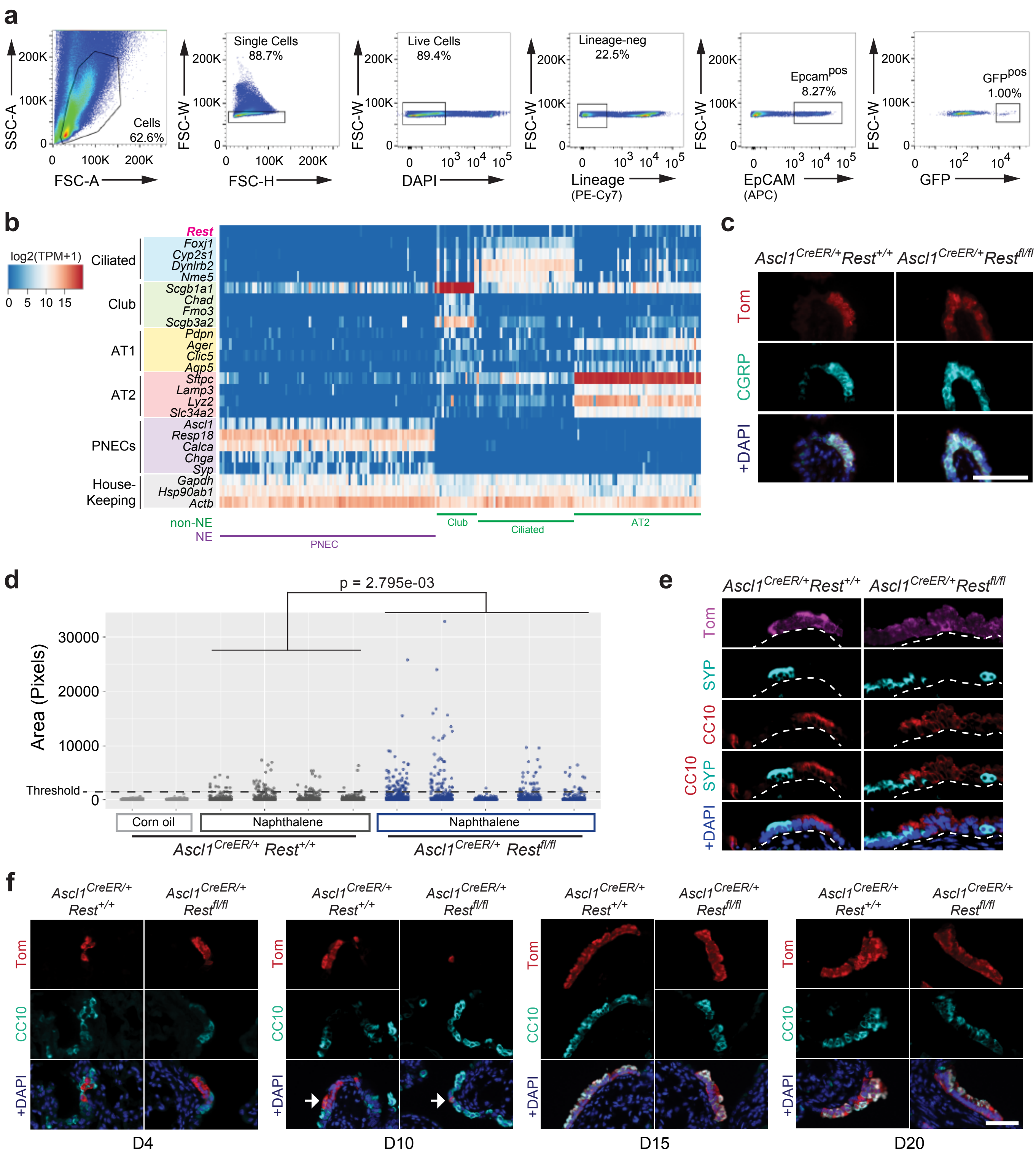


**Supplementary Figure 7: Loss of REST results in increased contribution of neuroendocrine cells to lung repair following injury**

**a,** Flow cytometry strategy to isolate pulmonary neuroendocrine cells (PNECs) and non-NE lung epithelial cells from the lungs of adult *Chga-GFP* mice. **b,** *Rest* expression from publicly-available single-cell RNA-seq dataset of mouse lung epithelial cells. **c,** Immunostaining for the Tomato reporter and the PNEC marker CGRP on lung sections from mice 4 weeks after treatment with tamoxifen but not naphthalene. DAPI stains DNA in blue. Scale bar, 50 μm. **d,** Quantification of normal neuroepithelial bodies (NEBs) and PNEC outgrowths after injury as in Fig. 4d in a second independent experiment (n=2 mice for corn oil control, 4 mice for naphthalene-treated *Rest*$^{+/+}$ and 5 mice for naphthalene-treated *Rest*$^{fl/fl}$). A Wilcoxon rank sum test was conducted on the combined PNEC outgrowths from all the mice in each respective group that are above the threshold size of the non-injured controls (n=80 for *Rest*$^{+/+}$,n=193 for *Rest*$^{fl/fl}$). **e,** Immunostaining for the Tomato reporter, the PNEC marker SYP and the club cell marker CC10 on section from lung lobes 3 weeks after injury. DAPI stains DNA in blue. White dotted lines demarcate basement membrane. Scale bar, 50 μm. Note: Tomato was stained on a consecutive section. **f,** Immunostaining for the Tomato reporter (red) and Club cell marker CC10 (cyan) on representative lung sections from mice at D4, D10, D15, D20 after naphthalene injury. DAPI stains DNA in blue. Scale bar, 50 μm. Source data are provided as a Source Data file.

# Supplementary Figure 8

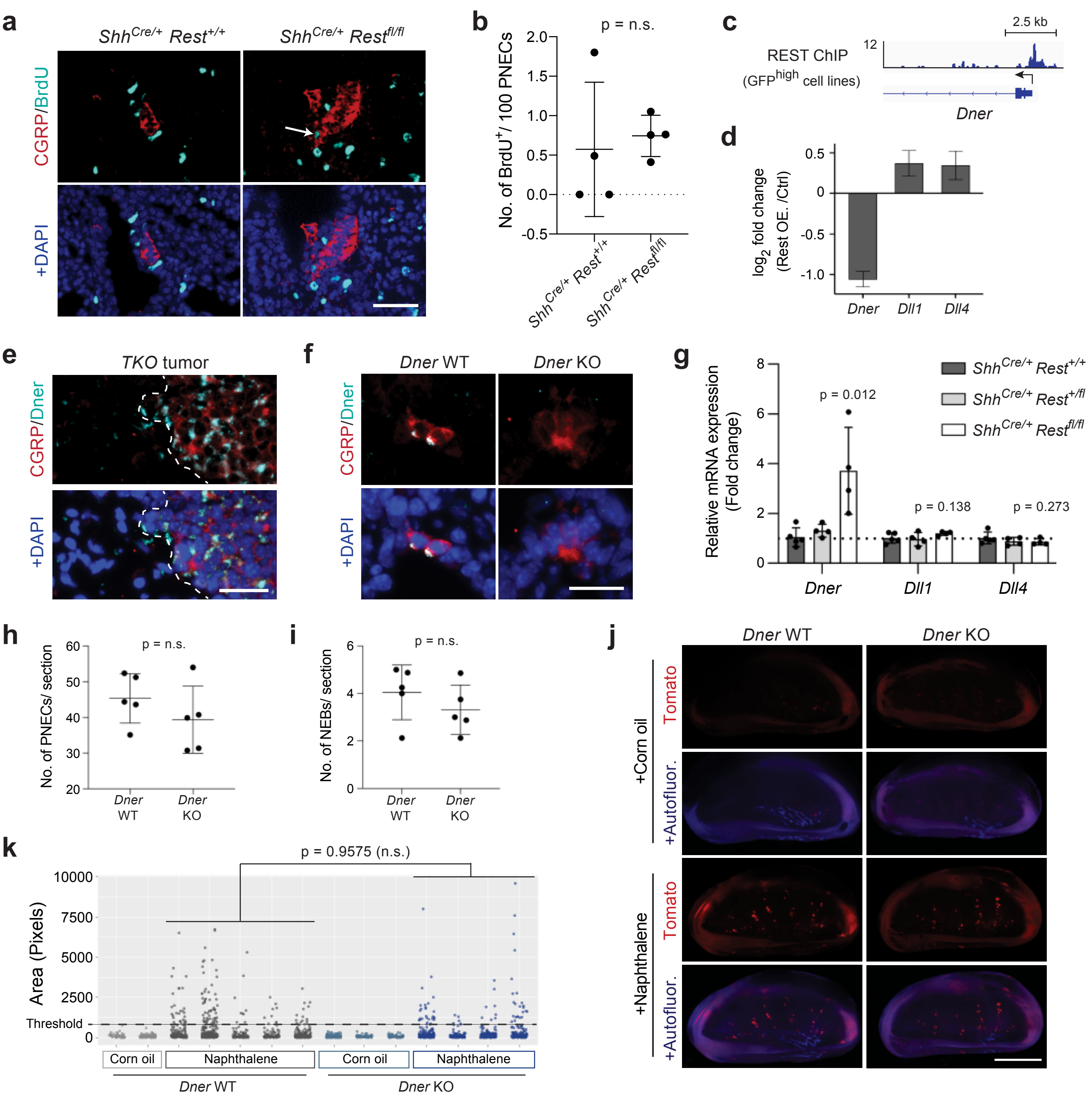


**Supplementary Figure 8: The atypical NOTCH ligand DNER does not control the neuroendocrine cell fate in mouse embryonic lungs**

**a,** Immunostaining for CGRP and the proliferation marker BrdU in E18.5 *Shh*$^{cre/+}$; *Rest*$^{+/+}$ or *Rest*$^{fl/fl}$ lungs. DAPI stains DNA in blue. White arrow identifies BrdU$^{+}$ pulmonary neuroendocrine cells (PNECs). Scale bar, 50 µm. **b,** Quantification of BrdU$^{+}$ PNECs (n=4 embryos per genotype, and 3 sections counted per embryo). Unpaired t-test. Data represented as mean ± s.d. **c,** ChIP-seq data showing REST binding near TSS of *Dner*. **d,** RNA-seq data for Notch ligands 5 days after REST overexpression (n=3). Error bars, log2 fold change estimate ± lfcSE. **e,** Immunostaining for CGRP and DNER in a representative *TKO* tumor. White dotted lines demarcate tumor border. DAPI stains DNA in blue. Scale bar, 25 µm. **f,** Immunostaining for CGRP and DNER in E18.5 *Dner* WT or *Dner* KO lungs. DAPI stains DNA in blue. Scale bar, 20 µm. **g,** Relative mRNA expression levels of Notch ligands in E18.5 *Shh*$^{cre/+}$; *Rest*$^{+/+}$, *Rest*$^{+/fl}$, or *Rest*$^{fl/fl}$ lungs (n=5 embryos for *Rest*$^{+/+}$ and 4 embryos for *Rest*$^{+/fl}$ and *Rest*$^{fl/fl}$). Unpaired t-test, data represented as mean ± s.d. **h,i,** Quantification of CGRP$^{+}$ PNECs (h) and neuroepithelial bodies (NEBs) (≥ 5 PNECs) (i) per lung section from each *Dner* WT or KO embryo (n=5 *Dner* WT embryos with 40 sections counted and 5 *Dner* KO embryos with 39 sections counted). Unpaired t-test, data represented as mean ± s.d. **j,k,** Representative whole-mount fluorescence images (j) of left lung lobes 3 weeks after injury. Scale bar, 5 mm. (k) Quantification of normal NEBs and PNEC outgrowths after injury as in (j) with each column representing a single mouse and each data point a Tomato-positive cluster in that mouse (n=2 mice for corn oil-treated *Dner* WT, 5 mice for naphthalene-treated *Dner* WT, 3 mice for corn oil-treated *Dner* KO, 4 mice for naphthalene-treated *Dner* KO). A Wilcoxon rank sum test was conducted on the combined PNEC outgrowths from all the mice in each respective group that are above the threshold size of non-injured controls (n=127 for *Dner* WT, n=68 for *Dner* KO). Source data are provided as a Source Data file.

# Supplementary Figure 9

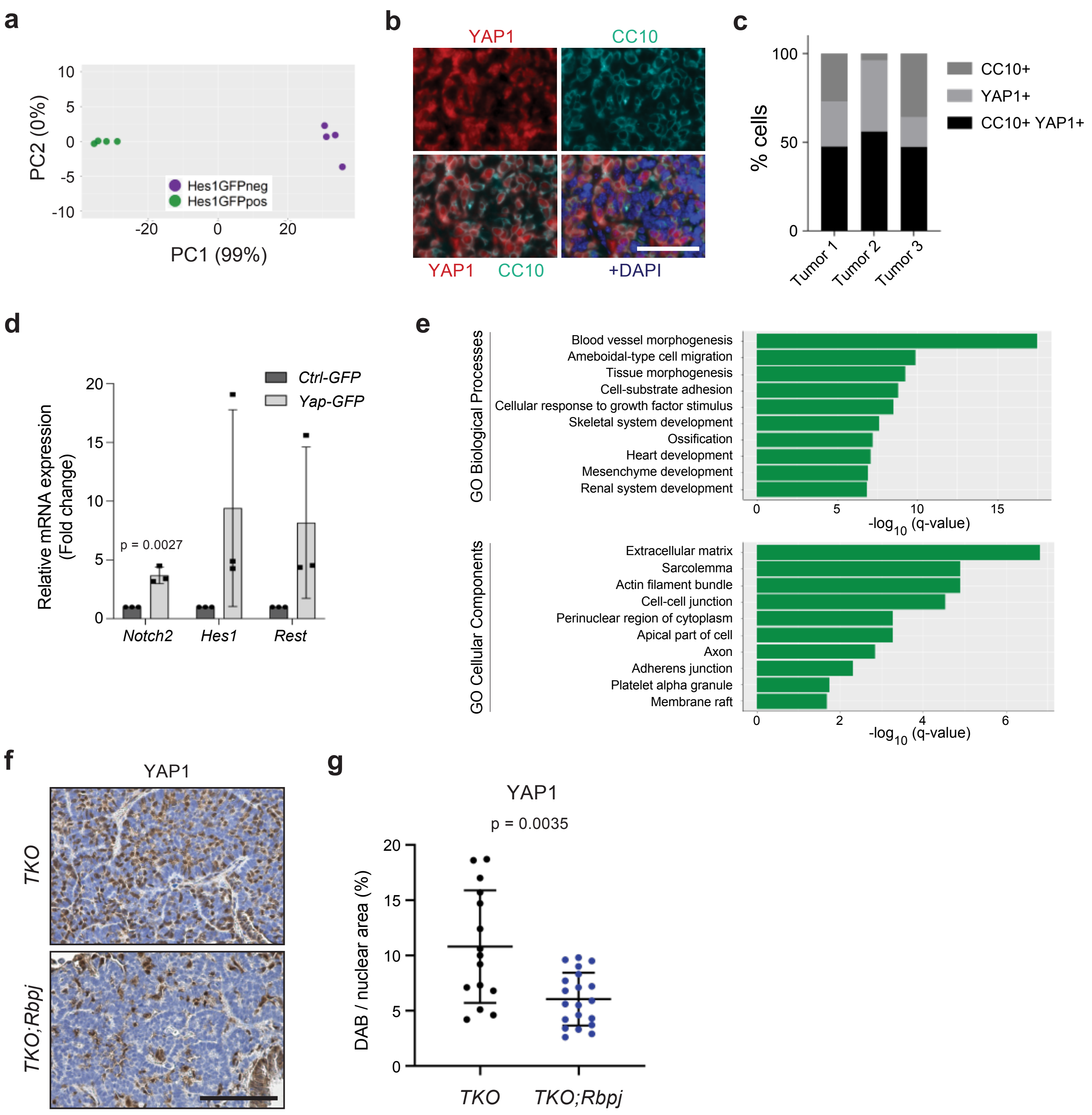


**Supplementary Figure 9: YAP1 expression promotes the non-neuroendocrine fate**

**a,** Principal component analysis (PCA) of ATAC-seq data comparing GFP$^{high}$ and GFP$^{neg}$ SCLC cells from the *TKO;Hes1*$^{GFP/+}$ mouse model (n=4 paired samples, 3 pairs of biological replicates and 1 technical replicate). **b,c,** (b) Representative immunofluorescence images for YAP1 expression (red) and CC10 (cyan) on a section from a *TKO;Hes1*$^{GFP/+}$ mouse SCLC tumor. DAPI stains DNA in blue. Scale bar, 50µm. (c) Quantification (%) of cells that are YAP1$^+$, CC10$^+$ or YAP1$^+$ CC10$^+$ in n=3 mouse tumors (as in b) shows that large proportion of YAP1$^+$ are CC10$^+$. **d,** Expression levels of *Notch2, Hes1*, *Rest* in neuroendocrine mouse KP1 SCLC cells 5 days after YAP1 overexpression by RT-qPCR (n=3). Unpaired t-test, data represented as mean ± s.d. **e,** Gene ontology (GO) enrichment of upregulated genes after 5 days of YAP1 overexpression in KP1 cells for Biological Processes and Cellular Components. **f,g,** (f) Representative immunohistochemistry images of YAP1 staining (brown signal) in *TKO* and *TKO;Rbpj*$^{fl/fl}$ tumors (hematoxylin counterstain). Scale bar, 100 µm. Quantification of YAP1 immunostaining (g) in tumor sections (n=15 tumors from 3 mice for *TKO* and 20 tumors from 4 mice for *TKO;Rbpj*$^{fl/fl}$). Unpaired t-test with Welch's correction, data represented as mean ± s.d. Source data are provided as a Source Data file.

# Supplementary Figure 10

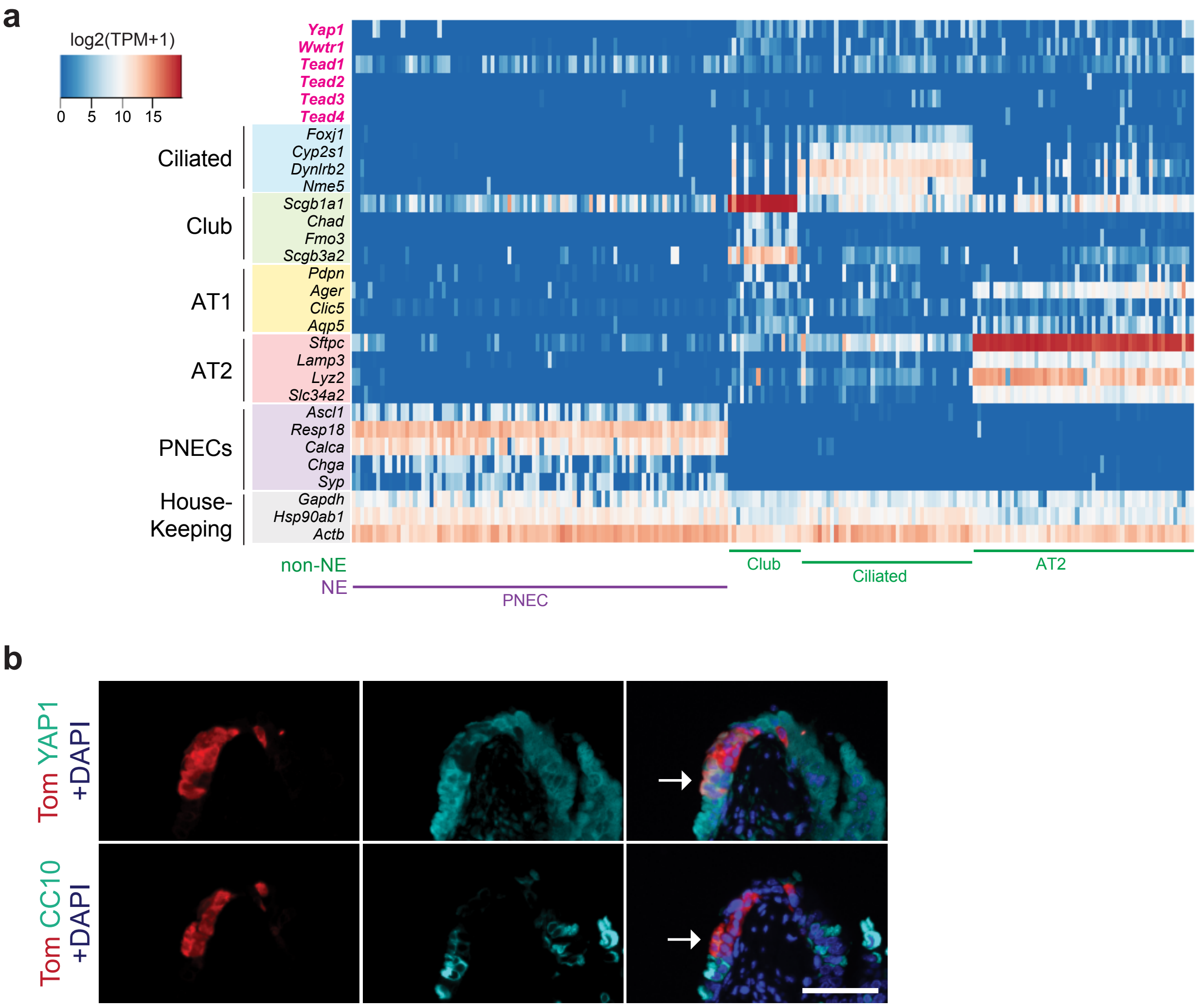


**Supplementary Figure 10:** ***Yap1*** **is mostly not expressed in pulmonary neuroendocrine cells except for a few rare cells** **a,** Heatmap of publicly-available single-cell RNA-seq data for the expression of *Yap1* and Hippo pathway members in PNECs and non-NE lung epithelial cells in normal mouse lung epithelial cells. **b,** Immunostaining for the Tomato reporter (red) and either Club cell marker CC10 or YAP1 (cyan) on consecutive lung sections from *an* $Ascl1^{CreER/+}$ $Rosa26^{LSL\text{-}Tom/LSL\text{-}Tom}$ mouse ($Yap1^{+/+}$) at day 10 after naphthalene injury. Representative images from one experiment. DAPI stains DNA in blue. White arrows pointing to transdifferentiated PNECs (expressing CC10 and tdTomato), in which YAP1 expression is detectable. DAPI stains DNA in blue. Scale bar, 50 µm.
