# Supplementary material for: A conserved YAP/Notch/REST network controls the neuroendocrine cell fate in the lungs
Source: Nat Commun. 2022 May 16;13:2690. doi: 10.1038/s41467-022-30416-2 (PMC9110333; doi:10.1038/s41467-022-30416-2)
Supplement: Supplementary file 3 — Description of Additional Supplementary Files [file 41467_2022_30416_MOESM3_ESM.docx]

**Description of Additional Supplementary Files**

**Supplementary Data 1:** Differential gene analysis comparing Hes1-GFP^high^ to Hes1-GFP^neg^ SCLC cells sorted fresh from tumors in mice determined by RNA-seq and Enrichr transcription factor analysis of downregulated genes.

**Supplementary Data 2:** Gene Ontology (GO) analysis of differentially expressed genes comparing Hes1-GFP^high^ to Hes1-GFP^neg^ SCLC cells sorted fresh from tumors in mice by Metascape.

**Supplementary Data 3:** Annotated peaks/targets of REST ChIP-seq in Hes1-GFP^high^ SCLC cell lines.

**Supplementary Data 4:** Differential gene analysis comparing REST-overexpressing to control KP1 mouse SCLC cells at 48 hours and 5 days timepoints determined by RNA-seq.

**Supplementary Data 5:** High-confidence REST and ASCL1 targets shortlisted by ChIP-seq and RNA-seq datasets.

**Supplementary Data 6:** Annotated peaks/targets of ASCL1 ChIP-seq in TKO tumors.

**Supplementary Data 7:** Differential gene analysis comparing sh*Ascl1* #1 or #2 to sh*GFP* control in KP1 mouse SCLC cells at Day 3 determined by RNA-seq. ASCL1 targets shortlisted by ASCL1 knockdown/ChIP-seq as well as a list of ASCL1 targets that overlap with REST targets.

**Supplementary Data 8:** Gene Ontology (GO) analysis of common ASCL1 targets identified by sh*Ascl1* #1 and #2 by Metascape.

**Supplementary Data 9:** Gene Ontology (GO) analysis of high confidence REST and ASCL1 targets by Metascape.

**Supplementary Data 10:** Differential gene analysis comparing *TKO;Rest^fl/fl^* non-NE cell lines to *TKO;WT (Rest^+/+^)* non-NE cell lines derived from tumors in mice determined by RNA-seq and Gene ontology (GO) analysis of upregulated genes by Metascape.

**Supplementary Data 11:** Enrichr transcription factor analysis on list of derepressed genes in *TKO;Rest^fl/fl^* non-NE cells not identified as high confidence REST targets.

**Supplementary Data 12:** HOMER analysis of known motifs on the differentially accessible peaks (ATAC-seq) in Hes1-GFP^high^ vs Hes1-GFP^neg^ SCLC cells.

**Supplementary Data 13:** Differential gene analysis comparing YAP1-overexpressing to control KP1 mouse SCLC cells at a 5-days timepoint determined by RNA-seq and gene ontology (GO) analysis on upregulated genes by Metascape.

**Supplementary Data 14:** Lists of primers used for genotyping, Fluidigm single-cell RT-qPCR, and RT-qPCR.

**Supplementary Data 15:** Sample list of NE and non-NE cell lines derived from *TKO;WT (Rest^+/+^)* or *TKO;Rest^fl/fl^* tumors.
