# Supplementary material for: A conserved YAP/Notch/REST network controls the neuroendocrine cell fate in the lungs
Source: Nat Commun. 2022 May 16;13:2690. doi: 10.1038/s41467-022-30416-2 (PMC9110333; doi:10.1038/s41467-022-30416-2)
Supplement: Supplementary file 19 — Reporting Summary [file 41467_2022_30416_MOESM19_ESM.pdf]

nature research

Corresponding author(s): Julien Sage

Last updated by author(s): Mar 4, 2022

Reporting Summary

Nature Research wishes to improve the reproducibility of the work that we publish. This form provides structure for consistency and transparency in reporting. For further information on Nature Research policies, see our Editorial Policies and the Editorial Policy Checklist.

Statistics

For all statistical analyses, confirm that the following items are present in the figure legend, table legend, main text, or Methods section.

n/a	Confirmed	
☐	☒	The exact sample size (n) for each experimental group/condition, given as a discrete number and unit of measurement
☐	☒	A statement on whether measurements were taken from distinct samples or whether the same sample was measured repeatedly
☐	☒	The statistical test(s) used AND whether they are one- or two-sided<br>*Only common tests should be described solely by name; describe more complex techniques in the Methods section.*
☐	☒	A description of all covariates tested
☐	☒	A description of any assumptions or corrections, such as tests of normality and adjustment for multiple comparisons
☐	☒	A full description of the statistical parameters including central tendency (e.g. means) or other basic estimates (e.g. regression coefficient) AND variation (e.g. standard deviation) or associated estimates of uncertainty (e.g. confidence intervals)
☐	☒	For null hypothesis testing, the test statistic (e.g. F, t, r) with confidence intervals, effect sizes, degrees of freedom and P value noted<br>*Give P values as exact values whenever suitable.*
☒	☐	For Bayesian analysis, information on the choice of priors and Markov chain Monte Carlo settings
☒	☐	For hierarchical and complex designs, identification of the appropriate level for tests and full reporting of outcomes
☒	☐	Estimates of effect sizes (e.g. Cohen's d, Pearson's r), indicating how they were calculated

Our web collection on statistics for biologists contains articles on many of the points above.

Software and code

Policy information about availability of computer code

Data collection	Flow cytometry data was collected using BD FACSDiva (BD Biosciences). Microscopy images were collected using the Keyence microscope with the BZ-X Viewer program version 1.3.1.1 and BZ-X Analyzer 1.4.0.1., Olympus VS120.
Data analysis	For image analysis: FIJI/ImageJ 2.0.0-rc-69/1.52p; Java 1.8.0_66, ImmunoRatio 1.0c, ImmunoMembrane 1.0i, Interactive H-watershed 1.2.1, HALO, Qupath 0.2.3<br>FACS analysis: FACSDiva software and FlowJo v10<br>Gene set enrichment analysis: GSEA 4.0.3<br>Gene Ontology: Metascape 3.5<br>For RNA-seq analysis: Salmon v0.8.2 and DESeq2 v1.28.1<br>For ChIP-seq analysis: BWA v0.7.17, Samtools v1.9 and Homer v4.10. Heatmap of binding sites: Deeptools 3.5.1<br>For ATAC-seq anaylsis: Homer v4.10, DESeq2 v1.30.1, Bowtie2 v2.3.4.1, Samtools v1.8, MACS2 v2.2.5<br>General: Graphpad Prism 8.4.1, R version 4.0.2, ggplot 3.3.2, Enhanced Volcano 1.6.0

For manuscripts utilizing custom algorithms or software that are central to the research but not yet described in published literature, software must be made available to editors and reviewers. We strongly encourage code deposition in a community repository (e.g. GitHub). See the Nature Research guidelines for submitting code & software for further information.

Data

Policy information about availability of data

All manuscripts must include a data availability statement. This statement should provide the following information, where applicable:

- Accession codes, unique identifiers, or web links for publicly available datasets
- A list of figures that have associated raw data
- A description of any restrictions on data availability

All data generated in the paper is available in the SuperSeries: GSE171919
which contains the following subseries:
TKO;Hes1GFP RNAseq GSE171914
TKO;Hes1GFP ATAC-seq GSE171917
TKO;Rest RNAseq GSE171916
Rest & Yap OE RNAseq GSE171915
Rest ChIPseq GSE171918
Ascl1 KD GSE185413

To review GEO accession GSE171919:
https://www.ncbi.nlm.nih.gov/geo/query/acc.cgi?acc=GSE171919

Single-cell RNAseq data of the lungs was downloaded from GSE136580: https://www.ncbi.nlm.nih.gov/geo/query/acc.cgi?acc=GSE136580

Source data are provided with this paper.

Field-specific reporting

Please select the one below that is the best fit for your research. If you are not sure, read the appropriate sections before making your selection.

[x] Life sciences [ ] Behavioural & social sciences [ ] Ecological, evolutionary & environmental sciences

For a reference copy of the document with all sections, see nature.com/documents/nr-reporting-summary-flat.pdf

Life sciences study design

All studies must disclose on these points even when the disclosure is negative.

Sample size	Sample sizes were determined by pilot experiments or previous experiments in an earlier publication based on the amount of intrinsic variability between mice or cells of the same genotype/condition. Lim JS, et al. Intratumoural heterogeneity generated by Notch signalling promotes small-cell lung cancer. Nature 545, 360-364 (2017).
Data exclusions	4 cell lines isolated from TKO;Rest fl/fl tumors were excluded from RNA-seq analysis because they were found to not have Rest properly deleted based on Rest mRNA levels.
Replication	All experiments were independently replicated at least twice or findings verified using another experimental/ orthogonal methods.
Randomization	Mice in injury studies were randomly assigned to control or naphthalene treatment. Randomization is not relevant for in vitro cell line experiments.
Blinding	No blinding of experiments was done as the same investigator designed and performed the experiments.

Reporting for specific materials, systems and methods

We require information from authors about some types of materials, experimental systems and methods used in many studies. Here, indicate whether each material, system or method listed is relevant to your study. If you are not sure if a list item applies to your research, read the appropriate section before selecting a response.

Materials & experimental systems

n/a	Involved in the study
	☒ Antibodies
	☒ Eukaryotic cell lines
☒	Palaeontology and archaeology
	☒ Animals and other organisms
☒	Human research participants
☒	Clinical data
☒	Dual use research of concern

Methods

n/a	Involved in the study
	☒ ChIP-seq
	☒ Flow cytometry
☒	MRI-based neuroimaging

Antibodies

Antibodies used

FACS antibodies: CD45-PE-Cy7 (eBioscience 25-0451-82, clone 30-F11, 1:100), CD31-PE-Cy7 (eBioscience 25-0311-82, clone 390, 1:100), TER-119-PE-Cy7 (eBioscience 25-5921-82, clone TER-119, 1:100), CD24-APC (eBioscience 17-0242-82, clone M1/69, 1:200), F4/80-PE-Cy7 (BioLegend 123114, clone BM8, 1:100), EpCAM-APC (eBioscience 17-5791-82, clone G8.8, 1:100), CD45-Pacific Blue (BioLegend 103126, clone 30-F11, 1:100), CD31-Pacific Blue (BioLegend 102422, clone 390, 1:100), TER-119-Pacific Blue (BioLegend 116232, clone TER-119, 1:100), CD24-PE-Cy7 (Biolegend 101821, clone M1/69, 1:200), ICAM1/CD54-PerCP-Cy5.5 (Biolegend 116123, clone YN1/1.7.4, 1:100), NCAM1/CD56-APC (R&D Systems FAB7820A, clone 809220, 1:50), NCAM1-Alexa Fluor 488 (R&D Systems FAB7820G, clone 809220, 1:50) and ICAM1-PE-CY7 (BioLegend 116122, clone YN1/1.7.4, 1:100).

IHC antibodies:
Hes1 (Cell signaling technology 11988, 1:200), Yap(D8H1X)XP® (Cell signaling technology 14074S, 1:200), CC10/Scgb1a1 (Santa Cruz sc-9772, 1:50), Syp (Neuromics MO20000), 1:200, CC10/Scgb1a1 (Millipore 07-623, 1:200), RFP/Tomato (Biosource MBS448092, 1:500), CGRP (Sigma-Aldrich C8198, 1:200), BrdU (BD Biosciences 347580, 1:50), Dner (R&D Systems AF2254, 1:50), RBP-J/RBPSUH (D10A4)XP® (Cell Signaling Technology 5313, 1:200), AGER/RAGE (R&D Systems MAB1179, 1:200), Alexa Fluor®488 donkey anti-goat IgG (Invitrogen A-11055, 1:200), Alexa Fluor®488 donkey anti-rabbit IgG (Invitrogen A-21206, 1:200), Alexa Fluor®594 donkey anti-rabbit IgG (Invitrogen A-21207, 1:200), Alexa Fluor®594 donkey anti-goat IgG (Invitrogen A-11058, 1:200), Alexa Fluor®594 donkey anti-rat IgG (Invitrogen A-21209, 1:200) and Alexa Fluor®488 donkey anti-mouse IgG (Invitrogen A-21202, 1:200).

ChIP antibodies:
Yap(D8H1X)XP® (Cell signaling technology 14074S, 0.14ug of antibody per 10 million cells) and Rest antibody (Millipore 17-641, 4ug of antibody per 10 million cells).

Immunoassay antibodies:
ASCL1 (Santa Cruz sc-374104, 1:200), YAP1 (D8H1X)XP® (Cell Signaling Technology 14074S, 1:2000), NOTCH2 (Cell Signaling Technology 5732, 1:2000), HES1 (Cell Signaling Technology 11988, 1:50), UCHL1 (Sigma-Aldrich HPA005993, 1:5000), and HSP90 (Cell Signaling Technology 4877, 1:5000).

Validation

The following antibodies were validated for flow cytometry of mouse cells by the manufacturer using respective positive control cells (e.g. mouse splenocytes for CD45) compared to isotype control antibodies:
CD45-PE-Cy7 (eBioscience 25-0451-82, clone 30-F11), CD31-PE-Cy7 (eBioscience 25-0311-82, clone 390), TER-119-PE-Cy7 (eBioscience 25-5921-82, clone TER-119), CD24-APC (eBioscience 17-0242-82, clone M1/69), F4/80-PE-Cy7 (BioLegend 123114, clone BM8), EpCAM-APC (eBioscience 17-5791-82, clone G8.8), CD45-Pacific Blue (BioLegend 103126, clone 30-F11), CD31-Pacific Blue (BioLegend 102422, clone 390), TER-119-Pacific Blue (BioLegend 116232, clone TER-119), CD24-PE-Cy7 (Biolegend 101821, clone M1/69), ICAM1/CD54-PerCP-Cy5.5 (Biolegend 116123, clone YN1/1.7.4), NCAM1/CD56-APC (R&D Systems FAB7820A, clone 809220), NCAM1-Alexa Fluor 488 (R&D Systems FAB7820G, clone 809220) and ICAM1-PE-CY7 (BioLegend 116122, clone YN1/1.7.4).

The following antibodies were validated by manufacturer for IHC /Immunoassay using positive control tissue sections, positive control cell line lysates, gene knockout/knockdown and overexpression experiments:
Hes1 (Cell signaling technology 11988), Yap(D8H1X)XP® (Cell signaling technology 14074S), CC10/Scgb1a1 (Santa Cruz sc-9772), Syp (Neuromics MO20000), CC10/Scgb1a1 (Millipore 07-623), RFP/Tomato (Biosource MBS448092), CGRP (Sigma-Aldrich C8198), BrdU (BD Biosciences 347580), Dner (R&D Systems AF2254), RBP-J/RBPSUH (D10A4)XP® (Cell Signaling Technology 5313), AGER/RAGE (R&D Systems MAB1179), ASCL1 (Santa Cruz sc-374104), NOTCH2 (Cell Signaling Technology 5732), UCHL1 (Sigma-Aldrich HPA005993), and HSP90 (Cell Signaling Technology 4877).

Yap, RBP-J, Dner antibodies were further validated in our KO mice.

The following antibodies were validated by manufacturer for ChIP by performing ChIP in positive control cell lines and testing for enrichment in known target regions via qPCR:
Yap(D8H1X)XP® (Cell signaling technology 14074S) and Rest antibody (Millipore 17-641).

Eukaryotic cell lines

Policy information about cell lines

Cell line source(s)

All cell lines were generated in-house from mouse tumors with the exception of 293T, which was purchased from ATCC.

Authentication	293T was authenticated via Short Tandem Repeat (STR) profiling (Genetica). Mouse cell lines were not authenticated, however we had performed RNA-seq on all the lines either in this study or previously.
Mycoplasma contamination	All cell lines tested negative for mycoplasma.
Commonly misidentified lines (See ICLAC register)	None.

Animals and other organisms

Policy information about studies involving animals; ARRIVE guidelines recommended for reporting animal research

Laboratory animals	Mouse lines used were the triple-knockout (TKO) SCLC mouse model bearing deletions of floxed (fl) alleles of p53, Rb, and p130 as previously described (PMID: 20406986), GFP reporter expressed from the endogenous Hes1 promoter Hes1GFP allele (PMID: 21991352), Tomato reporter Rosa26lox-stop-lox-tdTomato (JAX: 007909), GFP reporter under the control of Chga promoter Chga-GFP (inserted as multiple copies of BAC, also crossed to TKO but no Cre was introduced) (PMID: 27298335), floxed Yap1 allele Yap1-fl (PMID: 21376238), floxed Rest allele Rest-fl (also named RestGTi) (PMID: 26745185), floxed Rbpj allele Rbpj-fl (PMID:11967543), Cre recombinase knocked into Ascl1 locus Ascl1CreER allele (also named Ascl1CreERT2) (JAX: 012882) and Cre recombinase knocked into Shh locus ShhCre allele (also named ShhGFPcre, featuring Cre fused to GFP) 7(JAX: 005622). Dner KO mice were generated by the Stanford Transgenic, Knockout, and Tumor Model Center (TKTC) via injection of C57BL/6 mouse zygotes with sgRNAs and Cas9 targeting sites in intron 1 and 4, resulting in a deletion of exon 2 to 4 containing the delta/notch-like EGF repeat region.<br>For injury and in vivo SCLC tumor studies, 8 to 12 weeks old mice were used. For developmental studies, E18.5 embryos were collected. For all animal experiments, mice of both genders were used.
Wild animals	None.
Field-collected samples	None.
Ethics oversight	Mice were maintained according to practices prescribed by the NIH at Stanford's Research Animal Facility (protocol #13565). Additional accreditation of Stanford animal research facilities was provided by the Association for Assessment and Accreditation of Laboratory Animal Care (AAALAC).

Note that full information on the approval of the study protocol must also be provided in the manuscript.

ChIP-seq

Data deposition

- [x] Confirm that both raw and final processed data have been deposited in a public database such as GEO.
- [x] Confirm that you have deposited or provided access to graph files (e.g. BED files) for the called peaks.

Data access links<br>*May remain private before publication.*	GSE171918<br>See Data section above for link to access the SuperSeries.
Files in database submission	GSM5237429 BC11_Hes1GFPhigh_ChIP1<br>GSM5237430 BC13_Hes1GFPhigh_ChIP2<br>GSM5237431 BC14_Hes1GFPhigh_input1<br>GSM5237432 BC16_Hes1GFPhigh_input2<br>And corresponding TAG directories<br>Siginificant peaks are provided as a supplementary table.
Genome browser session (e.g. UCSC)	Dropbox link to the bam/bai/tdf files for viewing on IGV:<br>https://www.dropbox.com/sh/7rz7v9vrjq7ouvo/AACfFS4fjNEsSEJtMuX7-7rQa?dl=0

Methodology

Replicates	2 replicates
Sequencing depth	75 bp reads, single-end.<br>Sequencing depth/Mapped<br>L2V18DR-BC11-AGCATG_S9_R1_001.fastq.gz: 41293421/5477958<br>L2V18DR-BC13-CGTAGA_S10_R1_001.fastq.gz: 47997911/10784986<br>L2V18DR-BC14-TCAGAG_S11_R1_001.fastq.gz: 46390232/46009604<br>L2V18DR-BC16-TTGGCA_S12_R1_001.fastq.gz: 48986889/48593820
Antibodies	Rest antibody (Millipore 17-641) .
Peak calling parameters	ChIP data were aligned using BWA to the mouse genome mm10 using the default settings. Aligned sequences were then processed using HOMER (http://homer.ucsd.edu/homer/) using the default settings (TAG directories, finding peaks, and motif analysis).

Data quality	Total Upregulated in target vs. input: 1493 (92.331%) [log2fold>1, FDR<0.05]<br>Total Dn-regulated in target vs. input: 0 (0.000%) [log2fold<-1, FDR<0.05]
Software	BWA 0.7.17, Samtools 1.9 and HOMER 4.10.

Flow Cytometry

Plots

Confirm that:

- [x] The axis labels state the marker and fluorochrome used (e.g. CD4-FITC).
- [x] The axis scales are clearly visible. Include numbers along axes only for bottom left plot of group (a 'group' is an analysis of identical markers).
- [x] All plots are contour plots with outliers or pseudocolor plots.
- [x] A numerical value for number of cells or percentage (with statistics) is provided.

Methodology

Sample preparation	Tumors isolated from the lungs of TKO;Hes1GFP/+ mice approximately 6-7 months after tumor induction were pooled, finely chopped with a razor blade and digested in 6 ml of PBS with 120 µL of 100 mg/ml collagenase/dispase (Roche) for 45 min in a 37ºC shaker. The mixture was then cooled on ice before adding 15 µL of 1 mg/mL DNase (Sigma) for 5 min. Digested tissue was passed through a 40 µm filter, spun down, and resuspended in 1 mL of red blood cell lysis buffer (150 mM NH4Cl, 10 mM KHCO3, 0.1 mM EDTA) for 1.5 min. Cells were spun down, washed once in DMEM and resuspended in FACS buffer (10% BGS in PBS, 1 million cells per 100 ul). The resultant single-cell suspension was stained with the following FACS antibodies: CD45-PE-Cy7 (eBioscience 25-0451-82, clone 30-F11, 1:100), CD31-PE-Cy7 (eBioscience 25-0311-82, clone 390, 1:100), TER-119-PE-Cy7 (eBioscience 25-5921-82, clone TER-119, 1:100), CD24-APC (eBioscience 17-0242-82, clone M1/69, 1:200) or CD45-Pacific Blue (BioLegend 103126, clone 30-F11, 1:100), CD31-Pacific Blue (BioLegend 102422, clone 390, 1:100), TER-119-Pacific Blue (BioLegend 116232, clone TER-119, 1:100), CD24-PE-Cy7 (Biolegend 101821, clone M1/69, 1:200), ICAM1/CD54-PerCP-Cy5.5 (Biolegend 116123, clone YN1/1.7.4, 1:100), NCAM1/CD56-APC (R&D Systems FAB7820A, clone 809220, 1:50) and DAPI to label dead cells.<br>Lungs from Chga-GFP mice were digested and stained as above with an additional lineage negative selection marker F4/80-PE-Cy7 (BioLegend 123114, clone BM8, 1:100) and EpCAM-APC (eBioscience 17-5791-82, clone G8.8, 1:100) instead of CD24 as positive selection marker.<br>Tumors isolated from the lungs of TKO;Restfl/fl or TKO;Yap1fl/fl mice were digested in the same way but stained with the following FACS antibodies: CD45-Pacific Blue (BioLegend 103126, clone 30-F11, 1:100), CD31-Pacific Blue (BioLegend 102422, clone 390, 1:100), TER-119-Pacific Blue (BioLegend 116232, clone TER-119, 1:100), CD24-APC (eBioscience 17-0242-82, clone M1/69, 1:200), NCAM1-Alexa Fluor 488 (R&D Systems FAB7820G, clone 809220, 1:50) and ICAM1-PE-CY7 (BioLegend 116122, clone YN1/1.7.4, 1:100).<br><br>Tumors isolated from the lungs of TKO;Restfl/fl or TKO;Yap1fl/fl mice were digested in the same way but stained with the following FACS antibodies: CD45-Pacific Blue (BioLegend 103126, clone 30-F11, 1:100), CD31-Pacific Blue (BioLegend 102422, clone 390, 1:100), TER-119-Pacific Blue (BioLegend 116232, clone TER-119, 1:100), CD24-APC (eBioscience 17-0242-82, clone M1/69, 1:200), NCAM1-Alexa Fluor 488 (R&D Systems FAB7820G, clone 809220, 1:50) and ICAM1-PE-CY7 (BioLegend 116122, clone YN1/1.7.4, 1:100).
Instrument	BD FACSAria II
Software	FACSDiva software and FlowJo v10.
Cell population abundance	Purity of the sorted cells were determined by mRNA transcripts levels of key genes via qPCR/single-cell qPCR/bulk RNA-seq analysis and morphology.
Gating strategy	Refer to supplemental figures for gating strategies of the different experiments.<br>In general, debris were excluded by SSC-A vs FSC-A and singlets gated by FSC-W vs FSC-H. Live cells were gated based on DAPI dye. Cells then undergo lineage negative selection (to remove hematopoietic lineages) followed by lineage positive selection (either CD24 for cancer cells or Epcam for PNECs). Finally, the cells were then gated on expression levels of each experiment-specific markers.

- [x] Tick this box to confirm that a figure exemplifying the gating strategy is provided in the Supplementary Information.
